# Supplementary material for: Linear-nonlinear cascades capture synaptic dynamics
Source: PLoS Comput Biol. 2021 Mar 15;17(3):e1008013. doi: 10.1371/journal.pcbi.1008013 (PMC7993773; doi:10.1371/journal.pcbi.1008013)
Supplement: Attachment — Submitted filename: V02_RossbroichSTP_ResponseReviewers.pdf [file pcbi.1008013.s001.pdf]

Response to the reviewers

We thank the reviewers for their critical assessment of our work. We greatly appreciated that they saw that our paper "*...will certainly be interesting to many computational and experimental neuroscientists...*" (Reviewer 1) and that they felt that our work "... *proposes an noteworthy alternative to describe synaptic short-term dynamics*" (Reviewer 2). We found all of the reviewers' critiques were very constructive, and we feel that by incorporating the reviewers' insights, the manuscript is now much improved. In the following we address the reviewers' concerns point by point. Notably, we have included a novel figure (new Figure 8) and new results for comparing the model to experimental data.

Reviewer comments here are in blue, while our additions in the manuscript file are highlighted in red. Note that in this document, unlike in the manuscript, we utilize author-year citation format so as to make it easy for the reviewers to find the references.

Reviewer 1

We begin by addressing Reviewer 1's comments.

Point 1.1 The demonstrations for how the model can describe basic STD and STF, sub/supralinear facilitation, and facilitation latency are all clear and help motivate the model. However, it is less clear that the model actually outperforms previous models on real responses either to controlled stimulation in vitro or during natural activity in vivo. Adding model comparison along these lines (even for a few synapses) would help strengthen the paper (e.g. with publicly available data https://portal.brain-map.org/explore/connectivity/synaptic-physiology and comparing using publicly available code from previous models https://senselab.med.yale.edu/ModelDB/showmodel.cshtml?model=149914).

The reviewer makes here an important point, adding additional data analysis, that specifically addresses some claims would indeed strengthen the paper. As suggested, we have implemented a systematic comparison between the Spike Response Plasticity model and the Tsodyks-Markram model, in their capacity to describe the synaptic dynamics of real synapses in response to controlled stimulation. We have decided not to use the publicly available code for estimating the distribution of Tsodyks-Markram model parameters in order to have the same fitting procedure applied to both our model and this model. We refer the reviewer to the new Figure 8 and the additional text referring to it.

Point 1.2 Adding too much flexibility could mean that the model may over-fit synaptic dynamics (and more easily be influenced by long-term plasticity too). Some discussion or analysis of this issue and the potential effect on generalization would be valuable.

This is indeed an important point. We thank the reviewer to quite appropriately point this out as we have not explicitly discussed this important point. We have taken a modeling view that is influenced by the properties of statistical inference, where the number of parameters should be chosen to either avoid overfitting or to act a regularization factors in large numbers. We have clarified this point in a new paragraph from the Inference section:

> "We treat the number of basis function as well as the timescale (or shape) of the basis functions for efficacy and variance kernel as meta-parameters. Such meta-parameters are considered part of the fitting procedure, rather than a characteristic of the mechanistic model. We emphasize this point because, although we have parametrized the efficacy kernel as a sum of exponential decays (see Methods) each characterized by a specific timescale, we do not expect that any of these timescales match the timescale of a specific biological mechanism. One reason for this comes from the fact that it is possible to capture reasonably well a mono-exponential decay with a well chosen bi-exponential decay. Thus, a single biological timescale can be fitted by the appropriate combination of two timescales. Together, some heuristics can be applied as to the number and the choice of timescale that we expect to see in a particular system (e.g. timescales faster than 1 ms are stimulus artefacts and longer than 1 min would be long-term plasticity), but the choice of meta-parameters should be guided by the properties of statistical inference: choosing either a small number of well-spaced timescales to avoid overfitting, or a very large number of timescales so as to exploit the regularizing effect of numerous parameters (Gerstner et al. 2014, Richards et al. 2019, Advani et al. 2020)."

- Results, Inference section, 3d paragraph.

Furthermore, in the new manuscript, we have added an explicit comparison of TM and SRP models that touches upon the presence of overfitting. We found that the SRP model predicts better the response to protocols that were not used for fitting, despite having over twice the number of parameters. See new section and new Figure 8.

Point 1.3 The justification for the variance kernel is not entirely convincing. Having a highly flexible model of heteroskedasticity is nice in some ways, but the origins of variability will be less clear. For example, it seems like a fixed mean-variance relationship (approximately

Binomial) could explain the changes in CV in Fig 2/6. This structure (where \sigma_i = f(\mu_i)) also seems relatively straightforward within this model framework.

We thank the reviewer for this comment. The exposition of the stochastic version of our model did not fully establish the rationale for our model of the probabilistic elements in synaptic transmission. The reviewer is right that a fixed mean-variance relationship may capture these effects in a restricted dynamical regime. Our rationale was rather to expose a more detailed and therefore more flexible model first, and then possible simplification second. To this end, the new manuscript reads:

> "Synaptic transmission is inherently probabilistic. The variability associated with synaptic release depends intricately on stimulation history, creating a complex heteroskedasticity. Such changes in variability may be a direct reflection of the history-dependent changes in amplitudes. Although a fixed relationship between the mean amplitude and the variance of synaptic responses could be expected if the only source of variability were the a fixed number of equal-sized vesicles being randomly released with a given probability (Fuhrmann et al. 2002a), the relationship should depend on the dynamics of both the changing number of readily releasable vesicles and the changing probability with which they release (Loebel et al. 2013). In addition, other sources of variability such as the mode of release (He et al. 2007) or the size of vesicles (Soares et al. 2019a, Bekkers et al. 1990a) are present. Figure 2C illustrates one type of heteroskedasticity observed experimentally, whereby the variability increases through a stimulation train but only for the physiological calcium condition. To capture these transmission properties, we must establish a stochastic framework. Since the mechanisms underlying the dynamics of the variability of synaptic release are not known, we first constructed a flexible but complex model, and considered simplifications as special cases."

- Results, Stochastic properties, first paragraph

> "This stochastic model has two important special cases. The first is the case of constant variance, which is obtained by setting the variance kernel to zero. In this case the CV of releases will be inversely proportional to the mean given in Eq. 4, thus in agreement with experimental data in 2.5 mM Calcium (Fig. 2C). The other case corresponds to variability that is proportional to the mean. In this case, we assume that the dynamics of variability will follow the dynamics of the mean amplitude. For this, we set $k_\mu = k_\sigma$. Although both mean and variance are modeled with the same kernel, different baseline parmeters can give rise to different dynamics of the CV. Both these simplifications are of interest because they drastically reduce the number of parameters in the model."

- Results, Stochastic properties, 5th paragraph

Point 1.4 At the same time, both the kernel and fixed mean-variance models would miss the fact that part of the hetereoskedasticity is due to sequential sampling where y_j and y_j-1 are not independent given S. Some additional discussion of when and why you would want *such* a flexible model of variance would be helpful.

The reviewer brings up an interesting hypothesis, one that is novel to us. It is an interesting point since, mechanistically, a larger release may have been caused with a larger number of released vesicles and thus to a transient depletion that could be causing a negative autocorrelation function. As a consequence, we have directly tested this hypothesis and present the results in the revised manuscript. Overall, we find no such autocorrelation in the mossy fiber synapse. We refer the reviewer to the new Figure 8 for this point and the text in the new section on experimental data.

Point 1.5 Relation to Convolutional Neural Networks – It's not clear to me exactly what this section adds to the results beyond an analogy to deep learning. It seems that this is less of a "result" and more of a discussion point, one that is already made nicely in the paragraph around line 438. Is there something that I'm missing? Some novel way of thinking about synaptic dynamics across multiple inputs? If not it just seems to point out a possible extension of the model from Ujfalussy et al., and I would suggest removing or deemphasizing it in the results.

We thank the reviewer for the constructive criticism. Our goal with this section was to seek a formal (as opposed to informal) parallel with the nomenclature and terminology used in machine learning. We agree with the reviewer that it is much of a discussion point, but we think that the mapping we present is not entirely trivial (particularly the implementation of dropout-like mechanisms) and therefore consists of a result, albeit minor. To de-emphasize this result we have changed the title to focus on the linear-nonlinear structure rather than convolutional units. In addition, we have thoroughly revised this section (also as per **Point 2.5**), as we wanted to make clear one important finding from this is not a mere extension of Ujfalussy et al. as our findings indicate that neurons without dendritic processing should be conceived as a two-layer neural network. We refer the reviewer to the new section 'Relation with Convolutional Units'.

Minor Issues raised by Reviewer 1:

- The title could be more specific.

Thank you for the constructive criticism. We have completely changed the title and made it more specific.

- Abstract – it would be helpful to clarify that these are short-term dynamics

As suggested, we modified the first sentence in the abstract: “Short-term synaptic dynamics differ markedly across connections and strongly regulate how action potentials are being communicated.”

- Line 11: “the connectome” might be not be familiar to all readers. Could just say “anatomical/structural connectivity.”

We changed ‘the connectome’ to ‘structural connectivity’.

- Line 52 Typo: “can be inferred accurately with limited amount of experimental data”… should be “a limited” or “amounts”

We changed the expression to ‘(...) with limited amounts of experimental data (...)’

- Line 53 – “Our work also makes explicit that synaptic dynamics extend the information processing of dendritic integration by adding another layer of convolution combined with nonlinear readout…” The way this is worded makes it sound like a fact rather than a way of conceptualizing/modeling.

To clarify that this is a conceptualization of the model implications, we changed the sentence in question to: “Our modelling framework also suggests that synaptic dynamics can be conceptualized as an extension to the information processing of dendritic integration by adding another layer of convolution combined with nonlinear readout (...)”

- Fig 1 caption – could change “impulse response change in efficacy” to “efficacy kernel” for consistency.

As suggested, we changed “impulse response change in efficacy” to “efficacy kernel”

- “Sublinear and Supralinear Facilitation” ~line 135 – adding a brief overview of the TM model would be helpful here for readers who might not remember it exactly – e.g. state variables, parameters.

  Thank you for pointing this out. We have added the following statement:

  “This model captures the nonlinear interaction between depleting the readily releasable pool of vesicles (state variable R) and the probability of release (state variable u; see Methods for model description).”

  - Results, Line 142

- Line 167 – This paragraph is a bit unclear. Rather than “this variable”, it might be better to say “the baseline facilitation parameter”, throughout.

  We emphasized the difference between the *facilitation variable (u)* and the *baseline facilitation parameter (U)* in the TM model by introducing the respective notations (u and U) in the text and replacing ‘this variable’ with ‘u’.

- Fig 3 B/D/F – it might be helpful to normalize or change the axes on these plots so that B/D don’t appear range-restricted compared to F.

  We changed model parameters and y-axis scale of Figure 3 B/D/F so that model behavior does not appear range-restricted.

- Line 195 Typo: “burst of action potentials”

  Thank you. We changed to ‘burst of action potentials’

- Line 216 Typo: “Fig 3A” should be “Fig 2A”?

  Correct, Thank you. Changed to ‘Fig 2A’

- Line 291 Typo: “posterior distributions”

  Thank you. We corrected the typo.

- Line 342: “when the proof given by Paninski applies”… it’s not obvious to me that it does apply with a logistic nonlinearity and gamma noise model. It would be helpful to specify whether or not it does with the f(.) assumed in this paper. It also may be useful to point out in this section that mapping to a GLM does not guarantee identifiability/convergence (Zhao and Iyengar, Neural Comp 2010) or give any protection against model misspecification (Stevenson, Neural Comp 2018).

Thank you for the references and pointing out the need for clarification. We added the following section to the manuscript:

“In some similar models, the likelihood function is convex (Paninski 2004), but since this is not the case in general (Zhao and Iyengar, 2010), parameter inference must control for the robustness of solutions.”

- Line 450 of redlined manuscript

We have also added a reference to Stenson 2018.

- Line 453: “…this algorithmic similarity suggests that the linear-nonlinear structure of synaptic processing capabilities on neural and neuronal networks.” This sentence seems incomplete.

We changed the sentence to : “Yet, this algorithmic similarity suggests that a linear-nonlinear structure of synaptic processing capabilities is shared between neural and neuronal networks.”

- Line 457: “…can optimize information processing.” Not sure this claim is justified. Optimize how? What cost function?

We are sorry for the vague statement. We have added two references to support this claim: Naud and Sprekeler 2018, Payeur et al. 2020 and Keijser & Sprekeler 2020.

- Line 503: It would be helpful to spell out the gamma distribution (e.g. shape and scale parameters).

We add a few lines detailing the relationship between the shape and scale parameters and the gamma distribution mean and standard deviation. The equation for the gamma distribution that results is also added.

- Line 518: Should include what nonlinearity was actually used for f(.)

  We added the following sentence to the paragraph: '(...) and f(.) denotes the nonlinear (sigmoidal) readout'

Reviewer 2

Point 2.1 How well would the model perform when cross-validated over different sets of experiments, i.e., experiments with different presynaptic stimulation patterns? It would be curious to see how well the model matches with experimental data when fitted to one stimulation patterns and exposed to another.

Also, can the model be fitted to quantitatively reproduce the experimental data? The "Inference" section (starting on pg. 11) only unsatisfactorily answers this question. In particular, the data and model results shown in Fig. 2B and Fig 3F appear different in the snse that the efficacy at physiological calcium concentration appears to close to zero in the model. An overlay figure and a quantification comparing data and model would help to judge how well the model captures the data.

This is a fair and valid point, which echoes Point 1.1. In the present revision of the manuscript we have added experimental data with which we have fitted and tested the model with cross validation. We find that the model is able to predict with high accuracy the responses to stimulation protocols that were not used for fitting. See new Figure 8 and associated new text in the section on experimental data.

Point 2.2 Why would the authors assume that the mono-exponential decay time is know in the inference method? This is hardly realistic when starting from experimental data with irregular stimulation patterns, for example. Why is the decay time not included in the inference demonstration?

We thank the reviewer for this question. We realize that we have not properly explained our rationale for the choice of time scales, which we view as meta-parameters, not as indications of the presence of a biological mechanism at this particular timescale. We refer the reviewer to our answer in Point 1.2 for a description of the changes to the manuscript that were made to that effect. In addition to those changes, we have modified the text following the description of the model in results:

“Importantly, although k_μ can be formalized as a sum of exponentially decaying functions, the choice of basis function does not force a specific timescale onto the efficacy kernel. Instead, it is the relative weighting of different timescales that will be used to capture the effective timescales.”

-Results, ‘Deterministic Dynamics’

Point 2.3 The authors argue that the model does not require a change in its structure to capture sublinear, supralinear facilitation or delayed facilitation. The model is presented to be “more adaptable” compared to the Tsodyks-Markram model. I would argue that all complexity is put in the efficacy kernel which has to be adapted to capture the different short-term plasticity phenomenon. Difficult to judge which model is more flexible.

We concur with the reviewer that an important element of flexibility in the SRP model comes from the complexity of its kernels. As such, the kernels implement an added element of flexibility since they can contain an arbitrary number of timescales, whereas the original version of the TM model has only two. So if there are clearly 3 timescales in the data, the TM model will require an extension. We note however, that this is not the only element of flexibility as the SRP model contains two additional elements: a sigmoidal nonlinearity and a kernel to capture the dynamics of variability. Furthermore, we show that at least some of these novel elements make the SRP model more accurate to predict experimentally recorded synaptic responses. In our revised manuscript, we have attempted to clarify the distinct sources of added flexibility:

> “The SRP model presents three sources of added flexibility with respect to the well-established TM model, 1) a efficacy kernel with an arbitrary number of timescales, 2) a nonlinear readout with both supra- and sub-linear regimes, and 3) an additional kernel allowing for independent dynamics of variability.”

- Discussion, first paragraph

as well as the importance of overfitting when increasing kernel complexity (see **Point 1.2**).

Point 2.4 The introduction can do a better job in ad equately justifying the model proposed here. In particular, the argument presented between lines 13 and 21 is reductionist. No experimentalist or modeler would claim that the full extent of short-term plasticity dynamics is captured by classifying synapses as short-term facilitating or depressing based on paired-pulse ratios. Furthermore, it remains unclear what is referred to as “complex STP dynamics”, or “complex synapses”. The short-comings of existing modeling approaches and the motivation for the modeling approach taken can certainly be presented in a more nuanced way.

Thank you for the feedback, we have revised the introduction. Please see revised introduction.

Point 2.5 Not being an expert in convolutional neural networks, I had problems to follow the related section on page 13 (l. 351-384). Maybe this part of the manuscript text could be better tailored to a broader (non-CNN-expert) audience.

We thank the reviewer for communicating this. We have strongly reworked this section and refer the reviewer to the new section 'Relation to Convolutional Neural Network with Dropout'.

Minor Issues raised by Reviewer 2:

- p. 4, l. 100 : "..., a nonlinear readout is potentially more apt .... ". Is that something the reader should be able to understand at this point?

  We thank the reviewer for pointing out this ambiguous statement. We have revised the associated text, which now reads

  "Although some early studies have used a linear readout (Varela et al. 1997), synaptic dynamics invoke mechanisms with intrinsic nonlinearities, like the saturation of release probability or the fact that the number of vesicles cannot be negative. The readout, f(.), will capture the nonlinear progression of PSC amplitudes in response to periodic stimulation."

  -p. 4, l. 100

- p. 6, l.181 : Mention here that the CV dynamics will be considered later in the model framework. I was disappointed while reading that it was glanced over at this point of the manuscript.

  We added the following sentence to the paragraph: Later in this manuscript, we expand the modelling framework to account for probabilistic synaptic transmission and demonstrate that the modification of the baseline parameter similarly explains the experimentally observed changes in CV.

- Fig. 3 : Which panel in A and C corresponds to which case shown in B and D?

  In addition to color-coding the baseline parameter, U, we added a legend to the relevant panels in Figure 3.

- Fig. 4 : Panel D and E labels convey the same message and do not reflect what is shown in D.

We understand that the similar labelling of Panels D and E are confusing. In Panel E, a different stimulation protocol is used, in which the number of APs, rather than the timing of the control spike, is varied. To clarify the difference, we added experimental data redrawn from Neubrandt et al. (2018) to Panel E of Figure 4, and added an illustration of the stimulation setup used.

- p. 9, l. 216 : It should probably read “Fig. 2A” in the parenthesis.

  Correct, we changed the reference to ‘Fig 2A’

- Fig. 7 : It is not clear to me why C would indicate little correlation (“l.311”).? If D shows anti-correlation, C seems to hint to positive correlation.

  Thank you for pointing this out. There was a typo in the manuscript, we have corrected it to indeed mention a positive correlation.
